# The future of battery data and the state of health of lithium-ion batteries in automotive applications

**DOI:** 10.1038/s44172-024-00299-w

**Published:** 2024-11-19

**Authors:** Friedrich von Bülow, Felix Heinrich, William Arthur Paxton

**Affiliations:** 1 Volkswagen AG, Wolfsburg, Germany; 2 Volkswagen AG, Center of Excellence Battery, Salzgitter, Germany; 3 Volkswagen Group of America Inc., Innovation and Engineering Center California, Belmont, CA USA

**Keywords:** Batteries, Scientific data

## Abstract

Operational data of lithium-ion batteries from battery electric vehicles can be logged and used to model lithium-ion battery aging, i.e., the state of health. Here, we discuss future State of Health definitions, the use of data from battery production beyond production, the logging & aggregation of operational data and challenges of the State of Health in automotive applications. Our suggestions could improve data transfer efficiency and data storage costs.

## Introduction

Lithium-ion batteries (LIBs) are attracting increasing attention by media, customers, researchers, and industrials due to rising worldwide sales of new battery electric vehicles (BEVs) ^1,2^. Manufacturers of BEVs, known as original equipment manufacturers (OEMs), as well as LIB cell manufacturers & suppliers need to ensure that the battery of the BEV fulfills the warranty specifications (e.g.: USABC (US)^3^: 15 years and 1000 cycles; EUCAR (Europe)^4^: BEV life equals 150,000 km and 22–24 MWh; NEDO (Japan)^5^: 10–15 years and 1000–1500 cycles^6^). This is often accomplished by limiting the usable battery capacity through the battery management system (BMS) to accommodate for capacity loss over the lifetime of the battery. In this case, the total (gross) capacity of the battery is greater than the usable (net) battery capacity^7^.

When LIBs age their performance characteristics like the capacity and internal resistance change. For example, the aging-induced reduction of the capacity results in a decrease of the range of BEVs which is a key factor for customers. Commonly, the aging state of LIBs is called State of Health (SOH): the SOH compares the current state of the battery to the state of a new battery at its beginning of life (BOL). It depends on the usage and environmental conditions of the battery^8–10^. The SOH is also relevant for other battery applications like grid energy storage and electric trains^11,12^. The rising connectivity of machines in the context of the Internet of Things and Industry 4.0 also includes vehicle fleets so that real-world battery data from automotive applications become available on a large scale^13^.

Based on this situation, this paper contributes a re-evaluation on battery data and SOH with suggestions for the future development in the field, with a focus on automotive applications. Each of the suggestions is covered in a separate section of this paper covering different aspects of data, SOH, and battery.

**Fig. 1:**
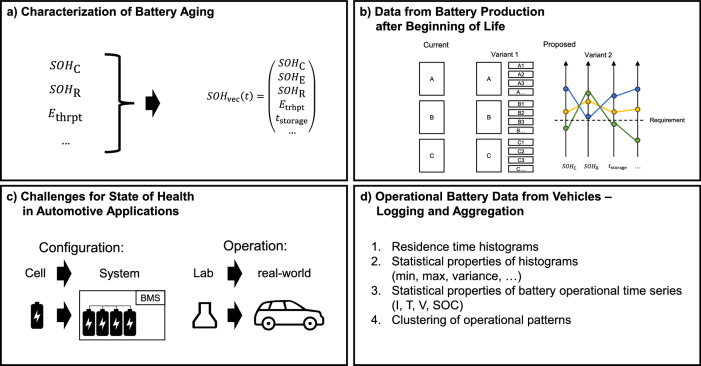
Illustration of the paper’s structure. **a** Characterization of Battey Aging.** b** Data from Battery Production after Beginning of Life. **c** Challenges for State of Health in Automotive Applications. **d** Operational Battery Data from Vehicles – Logging and Aggregation.

The remainder of this paper is structured as follows and illustrated by Fig. 1:

- The most frequently used SOH definition is the relative capacity, but also the relative storable energy content and the relative internal resistance are used^13^. For a more descriptive quantification and characterization of the SOH, we discuss the suitability of these definitions and propose another variant. This could lead to an improvement of SOH estimation and SOH forecasting methods.
- Given the task split of battery cell manufacturers and BEV manufacturers, data sharing beyond general cell specification sheets is currently uncommon due to silo-thinking. We argue that it would be beneficial to expand battery-cell classification after cell production and to use data from battery manufacturing beyond cell production.
- Research on SOH estimation, SOH forecasting, and remaining useful life (RUL) prediction has mostly been focused on data from LIB cells operated in the laboratory setting. However, in automotive applications, LIB systems combining hundreds (sometimes thousands) of cells operated under real-world conditions are subject to customers habits. We describe these differences in the operational domain (laboratory vs. real world) and the battery configuration (cell vs. system). Also, we provide examples of current challenges.
- When intending to conduct research on operational battery data, i.e., time-series data of current, temperature, voltage, and state of charge (SOC) from BEVs, suitable data logging, storage, and potentially aggregation need to be considered with the constraints of cost and mobile connectivity.

Operational data of LIBs from BEVs can be logged and used to model LIB aging, i.e., the SOH. Here, we discuss alternative SOH definitions which could reduce ambiguity in battery research. We also discuss the use of data from battery production beyond production itself, the logging and aggregation of operational data and challenges of the SOH in automotive applications. Our suggestions could improve data transfer efficiency and data storage costs.

## Minimal viable characterization of battery aging

The SOH compares the current state of the battery to the state of a new battery at its BOL^8–10^. The SOH can be defined differently depending on the view point, e.g., of a manufacturer or battery user^10^. Proposed state variables for the definition of the SOH that change with aging are: the number of charging/discharging cycles^14^, the voltage change caused by the load of a power or current profile^15^, AC impedance, self-discharge rate, and power density^8,16,17^. When using the term SOH of a battery, very often it is synonymously used for the ${{SOH}}_{{{\rm{C}}}}$, which is the relative capacity^18^:

1 $${{SOH}}_{{{\rm{C}}}}\left(t\right)=\frac{C\left(t\right)}{{C}_{{{\rm{nom}}}}}$$

with the remaining capacity $C\left(t\right)$ relative to the nominal capacity ${C}_{{{\rm{nom}}}}$ which is specified by the battery manufacturer or OEM.

The relative storable energy (${{SOH}}_{{\mbox{E}}}$) is correlated to the ${{SOH}}_{{{\rm{C}}}}$ by the voltage^19^:

2 $${{SOH}}_{{{\rm{E}}}}(t)=\frac{{E}_{\max }(t)}{{E}_{{{\rm{nom}}}}}$$

with the total energy ${E}_{\max }(t)$ and the nominal total energy ${E}_{{{\rm{nom}}}}$. The United Nations (UN) Global Technical Regulation (GTR) No. 22^20^ accelerates the importance of the energy and not the capacity as a measure of the SOH for developers, users, vendors, and buyers of BEVs by defining the usable battery energy (UBE), i.e., the net energy of the battery. The net energy is more relevant in practice to users of BEVs than the gross energy as it reflects the accessible and usable energy of the battery. The corresponding definition of a UBE-based ${{SOH}}_{{{\rm{UBE}}}}(t)$ similar to Eq. (2) but with explicit net energy:

3 $${{SOH}}_{{{\rm{UBE}}}}\left(t\right)=\frac{{{UBE}}_{\max }\left(t\right)}{{{UBE}}_{{{\rm{nom}}}}}=\frac{{E}_{\max ,{{\rm{net}}}}(t)}{{E}_{{{\rm{nom}}},{{\rm{net}}}}}$$

has been used very rarely, e.g., by Weng et al.^21^. Compared to the ${{SOH}}_{{{\rm{C}}}}$ fewer research works use the ${{SOH}}_{{{\rm{E}}}}$ and ${{SOH}}_{{{\rm{UBE}}}}$ health metrics. This may become problematic in case of a potential disconnect of academia from the upcoming standards and SOH definitions used in industrial and automotive applications.

Less frequently used is the relative resistance (*SOH*_R_)^22–24^:

4 $${{SOH}}_{{{\rm{R}}}}\left(t\right)=\frac{R\left(t\right)}{{R}_{{{\rm{nom}}}}}$$

with the current internal ohmic resistance $R\left(t\right)$ and the nominal internal ohmic resistance ${R}_{{{\rm{nom}}}}$. An overview of further *SOH*_R_ definitions is presented in Supplementary Note 1. For further differences and advantages of ${{SOH}}_{{{\rm{C}}}}$, ${{SOH}}_{{\mbox{E}}}$, and ${{SOH}}_{{\mbox{R}}}$ see Chapter 2.1.3 in ref. ^13^.

Unfortunately, these definitions may falter under certain scenarios which may lead to an ambiguous description of the SOH. This is illustrated by two examples: example 1 considers two batteries that have experienced different operations in the past as shown in Fig. 1. One battery has experienced only calendar aging (blue), and the other only cyclic aging (black). At a certain point in time, using the ${{SOH}}_{{\mbox{C}}}$ definition they are equivalent, but the batteries will likely continue on a different trajectory even when operated identically (green dotted). As a similar example, Hartmann et al.^25^ stored two cells for 150 days at marginally different conditions (100% SOC, 45 °C vs. 80% SOC, 60 °C). Afterwards, the capacity of both cells was similar, but during following fast-charging one cell experienced sudden death (rapid capacity degradation). These examples underline that the description of the ${{SOH}}_{{{\rm{C}}}}$ fails to account for different aging mechanisms triggered throughout the operations in the past.

Example 2 considers a single battery that is subjected to high-current cycling and capacity loss, but then recovers capacity after a long rest period^26–28^. As depicted in Fig. 3, this means the battery has the same ${{SOH}}_{{\mbox{C}}}$ more than once (red opaque area) during its lifetime. This means the description of the SOH fails in this ambiguous time where ${{SOH}}_{{\mbox{C}}}$ values are not decreasing monotonically.

These examples show the limitations of relying on a single scalar, like the relative capacity (${{SOH}}_{{\mbox{C}}}$), to describe the SOH of a battery as noted by Baure et al.^29^, Rogge et al.^30^, and Hartmann et al.^25^. Also, Weng et al.^21^ note that similarly even accurate single point measurements of the SOH are not sufficient to predict the RUL.

When analyzing the meaning of “state of health”, and specifically “state”, definitions and perceptions of this term come from control engineering^31,32^, thermodynamics^33^, Markov decision processes (MDPs)^34^, and reinforcement learning^34^. These state variables are “a set of variables that completely describe a system”^32^. In other words “the state must include information about all aspects of the past […] that make a difference for the future”^34^. This is also called Markov property^34^. States “always take on the same value when the state of the system is the same again”^33^. In other words, state variables only depend on the system’s state and should be path independent, i.e., they do not depend on the way how a state was reached^33^. A common example in thermodynamics is the two independent state variables temperature and pressure of pure liquid water (one component, one phase). They can be varied independently as degrees of freedom of pure liquid water to describe the state of the system as visible in the corresponding phase diagram^35,36^.

When transferring this aspiration to completely describe a battery system regarding the progress of its aging mechanisms and overall state of aging (SOA), this may be very ambitious given the complexity of battery aging. Still, capturing the SOA more precisely than only by a single variable like the relative capacity or the relative resistance seems advisable. It seems that a good balance of high level, e.g., on operation level, and low-level states, e.g., on the level of aging mechanisms, is advantageous.

**Fig. 2:**
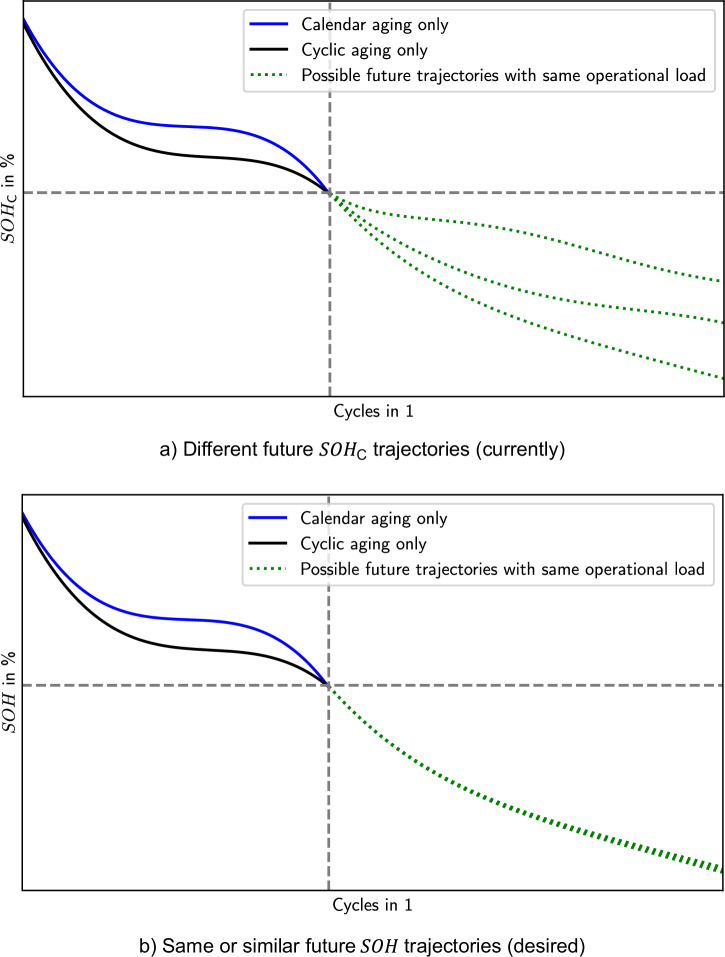
Example 1 of two batteries with different operations, but same *SOH*_C_. **a** Different future *SOH*_C_ trajectories (currently): *SOH*_C_ trajectories of two batteries that either first face only calendar (blue) or cyclic aging (black), respectively, but have the same ${{SOH}}_{{\mbox{C}}}$ at a certain point (gray dashed). From there on, they are cycled with the same operational load. **b** Same or similar future ${{SOH}}$ trajectories (desired): the same operational load leads to different future ${{SOH}}_{{\mbox{C}}}$ trajectories (green dotted) despite the same operational load. However, the same future operational load should lead to the same or very similar future ${SOH}$ trajectories (green dotted). Thus, a more suitable SOH definition should be selected﻿ (illustrative examples).

**Fig. 3:**
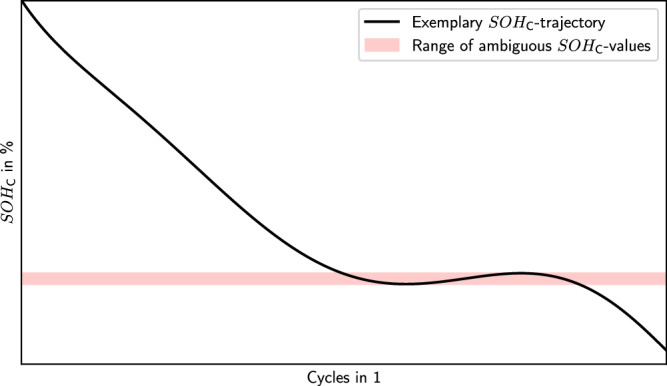
Example 2 of a battery with capacity recovery effect and ambiguous *SOH*_C_. *SOH*_C_ trajectory with capacity recovery effect having ambiguous ${{SOH}}_{{\mbox{C}}}$ in the read opaque area (illustrative example).

In essence, a “state of health” should capture the different battery aging paths and the relevant complexity of the battery’s state while avoiding too much simplicity. This aims at improving the distinguishability of the SOH of batteries with the same relative capacity, like those in the examples in Figs. 2 and 3.

How could the aging state be described more precisely? The idea of “fusing”^17^ different SOH variants/definitions like ${{SOH}}_{{\mbox{C}}}$, ${{SOH}}_{{\mbox{E}}}$, and ${{SOH}}_{{\mbox{R}}}$ has already been brought up by Saxena et al.^17^, but has not been applied widely. Fusion, in our interpretation, refers to a single scalar built from several scalars, e.g., by applying an arithmetic, harmonic, or geometric mean as defined in Supplementary Note 2^18^. However, when calculating the mean, some scaling, e.g., min–max normalization to the range $[{\mathrm{0,1}}]$ or usage of only relative values, might be sensible to balance the importance of all scalars. Furthermore, a single scalar compresses information which eases interpretability and comparability but hardens the distinguishability of the health of different battery cells. The latter is a limitation we would like to minimize.

We promote a more precise description of SOH in the form of a vector. The objective is to concatenate several scalars to one vector so that no information is aggregated or compressed:

5 $${{SOH}}_{{{\rm{vec}}}}\left(t\right)=\left(\begin{array}{c}{{SOH}}_{{\mbox{C}}}\\ {{SOH}}_{{\mbox{E}}}\\ \begin{array}{c}{{SOH}}_{{\mbox{R}}}\\ {E}_{{{\rm{trhpt}}}}\\ \ldots \end{array}\end{array}\right)$$

The vector may consist of the currently dominant ${{SOH}}_{{\mbox{C}}}$, ${{SOH}}_{{\mbox{E}}}$, and ${{SOH}}_{{\mbox{R}}}$, but also application-dependent parameters that contain information about the operational context. For a BEV this could include the mileage in kilometers and the number of equivalent full cycles. More application-independent parameters like the total energy throughput ${E}_{{{\rm{trhpt}}}}$ as well as distributional information on the SOC, voltage, and temperature during rest phases may indicate to which extend calendar aging mechanisms have been triggered. The same applies for cyclic aging mechanisms characterized by distributional information on the SOC, voltage, temperature, and current^19^. Including the share of rest time in the last X weeks and months may make sense to account for capacity recovery effects. Furthermore, harmful events like exceeding certain temperatures could be counted. Other examples can be found in the EU battery passport data attributes on battery “performance and durability”^37^.

Also, new battery materials may require a new or a more precise description of the SOA because of new different aging mechanisms. In this case again, capacity and resistance may not be sufficient to describe the SOH.

It might be sensible to store multiple capacity values obtained at different temperatures and different charging profiles as these variables influence the measured capacity. This would lead to a vector or matrix of capacities in which each scalar gets updated once a new capacity measurement at that temperature and charging profile gets measured during regular customer operation. This could tackle the problem that capacity measurements via reference cycles are more difficult to obtain in real-world BEV fleet operation than in the laboratory. Current challenges are time-consuming measurements and expensive test equipment with limited availablility^38–40^. A further improvement would be a method to make capacities comparable that have been determined under different conditions.

**Fig. 4:**
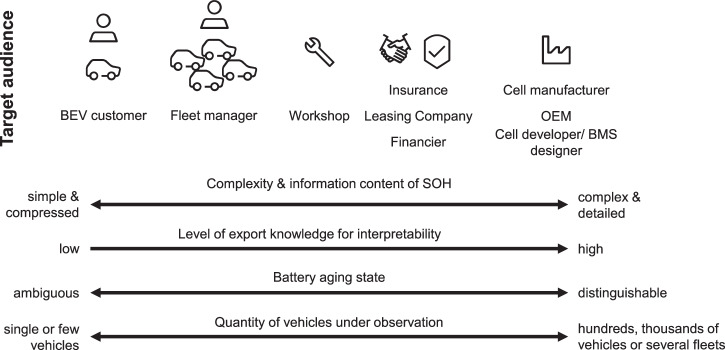
Target groups of different SOH definitions. Not every SOH definition may be suitable for all target groups interested in the battery state. Four possible dimensions with their characteristics are displayed. BEV battery electric vehicle, OEM original equipment manufacturer, BMS battery management system, SOH state of health.

Figure 4 visualizes different target audience groups and characteristics of a suitable SOH definition. On OEM side the BMS designers might be more interested in the influence of voltage margins in the BMS on the SOH, while the cell designers look into path dependency and aging mechanisms. The cell manufacturer is often concerned about cell quality and sudden deaths. In contrast, a leasing company, financier of BEVs, and insurance may be interested in the future value of a battery.

An SOH defined in vector form is certainly less easy to interpret for BEV customers or fleet managers. Most likely it will be difficult for them to retrieve operationally relevant characteristics like a standardized maximum range at fixed unpredictable variables, like road conditions, wind, and small energy consumers, from an SOH vector. Thus, the target group for the vector-based SOH would not be the BEV customer or a fleet manager. However, for methods of SOH estimation, SOH forecasting, and RUL prediction, a more elaborate and precise characterization of the SOH may be helpful to better distinguish different battery states from each other. This could also benefit BEV customers or a fleet managers if the methods perform better on a vector-based SOH, even though they keep only visible access to the ${{SOH}}_{{\mbox{C}}}$ or ${{SOH}}_{{\mbox{E}}}$.

## Using data from battery production after the beginning of life

Currently, at the end of battery cell production cells do not have equal properties due to the variety of production process steps and impurities in cells’ raw materials^41–44^. These initial cell-to-cell variations are coupled when the cells are assembled in a battery system. Then cell-to-cell variations of the capacity and impedance result in heterogeneous cell currents causing different heat generation of the cells. Then temperature gradients between cells can cause heat transfer to adjacent cells^45,46^. Overall, this cell-to-cell variance of aging stress factors leads to different SOH trajectories during battery life^47–49^. Despite, battery cells are often classified by the cell manufacturer into an ordinal scale of only three groups (grade A, B, and C)^50,51^. Heimes et al.^52^ also indicate three classes on page 21 in the center figure. However, there is no overall standard for this ABC classification. Every battery cell manufacturer may have its own regulations. Some cell manufacturers distinguish still fully functional grade-B and grade-C cells from grade-A cells by a higher storage time in a warehouse of several months^50^. However, the storage duration, storage temperature, and storage SOC are not further distinguished, even though it is known that they influence calendar aging^53,54^.

**Fig. 5:**
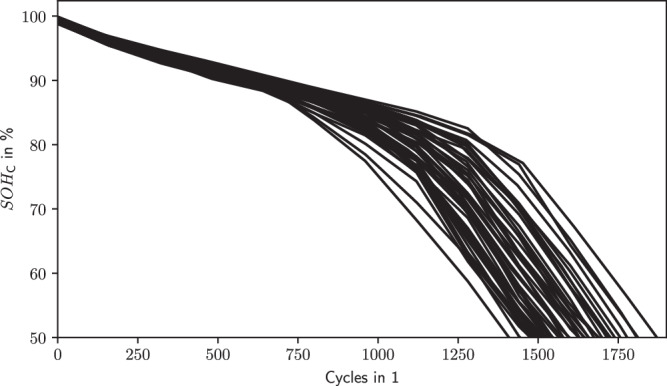
Increasing variability of *SOH*_C_ trajectories with the same initial grading. *SOH*_C_ trajectories of battery cells are graded into group C (data from ref. ^55^, plotted only ${{SOH}}_{{\mbox{C}}}\ge 50 \%$, data preprocessing applied is same as in previous works^13,85,86^).

Another example is the battery data set presented by Sauer et al.^55^ which uses 48 nominally identical battery cells. The cells were graded into group C from the cell manufacturer and are drawn from the same production lot. However, despite this and cycling with the same operational protocol for charging, discharging, and rest, the initially quite small variability of the capacity increases from the mid-life at around 800–1000 cycles onwards as shown in Fig. 5. This means that there was more variance regarding the health state of the battery cells initially than captured by the grouping into group C and the ${{SOH}}_{{\mbox{C}}}$.

Given the problem of precise battery cell grading after production, we want to initiate a discussion of an adapted grading system to end silo-thinking of battery cell manufacturing and operation: we propose either maintaining ordinal grading, but introducing more groups, or with a non-ordinal, but continuous grading using ratio scales. Both can be seen as an extension of the ordinal binning of the current grading system into A, B, and C. This shall enable a more precise characterization of battery cells, regardless of making or buying batteries. In the past, OEMs have mostly bought batteries for their HEVs or BEVs from 3rd party battery cell or module manufacturers. However, many OEMs strategically shift towards investing into in-house battery production^56^, which often is run as a subsidiary company. Regardless in both situations, make-or-buy, data exchange needs to be standardized given large organizations and complex processes.

**Fig. 6:**
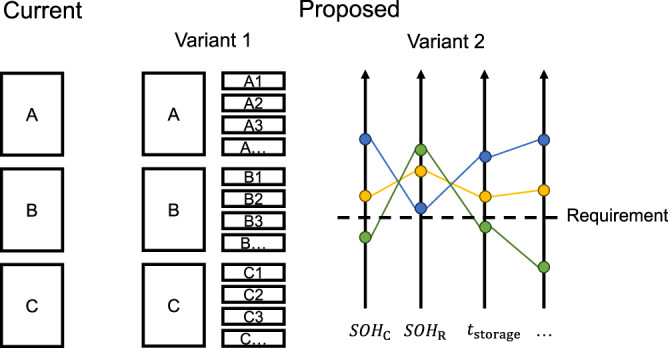
Possible evolution of battery cell grading system. Currently, battery cells are graded into three groups A, B, and C (right). We propose two variants: (1) a formation of subgroups like A1, A2, … corresponding to group A and (2) a multivariate grading which could be visualized by a (flattened) radar chart.

Variant 1 in Fig. 6 is an ordinal grading system, but more precise due to the subgroups. For example, B1 could contain cells with a storage time ${t}_{{{\rm{storage}}}}$ smaller than 1 month (${t}_{{{\rm{storage}}}}\le 1\,{{\rm{month}}}$), an average storage temperature of 15–20 °C (${\varnothing T}_{{{\rm{storage}}}}\in (15\,^{\circ}\, {{\rm{C}}},\,20\,^{\circ}\, {{\rm{C}}}]$), and an average storage SOC of 45%–55% (${\varnothing {SOC}}_{{{\rm{storage}}}}\in (45\% ,\,55\% ]$). Accordingly, another group is named B2 with $1\,{{\rm{month}}}\, < {t}_{{{\rm{storage}}}}\le 6\,{{\rm{month}}}$ and the other two conditions in the same range.

Variant 2 in Fig. 6 is a continuous grading system based on several battery health measures. As discussed in the previous section, potentially more characteristics of a battery cell than capacity and internal resistance are necessary to describe the SOH of a battery. This also applies to new battery cells after production. Figure 6 uses the display style “parallel coordinates”, but also a radar chart is possible to compare battery cells and batches of battery cells with each other relatively or with an absolute required specification.

This leads to the question how data from the battery production process can be used to characterize a battery cell. Currently, we observe silo-thinking/analysis: data from battery cell production processes are used to optimize and analyze the battery cell production process steps. Data from battery operation in the laboratory and real-world applications are used in the context of battery operation. We imagine that data from battery cell production can be used to characterize a battery cell (for more information on the battery production steps consult^52^). Data from battery cell production could originate from end-of-line tests (end-of-line is sometimes abbreviated as EoL or EOL. However, it is not abbreviated in this work due to the ambiguity with the acronym EOL for end-of-life). Contemplable end-of-line tests are pulse tests (e.g., direct current internal resistance test), open-circuit voltage tests, and self-discharge tests. But also data about the raw material batch and deviations from the desired process parameter (time, temperature, pressure, material, etc.) could indicate inadequate production batches regarding aging. Nevertheless, such data can also be advantageously used in the operation of the same battery cells as prior knowledge on the cells. For example, Baumhofer et al.^42^ found the initial pulse resistance to correlate with the cell life. Schindler et al.^48^ point out the applicability of differential voltage analysis and differential capacity analysis (DCA) to determine differences in the active material composition correlating with the trajectory of the cells’ capacities. Of course, this requires the sharing of such more detailed data on the cells and their state at the end-of-line by the cell manufacturer to the battery buyer, like academic institutions or BEV manufacturers. Regarding the use of battery cells in 2nd life applications, Rufino Júnior^57^ mentions an obstacle to intermediary companies who are trading 2nd life batteries with variations of battery cells. The mentioned data could reduce this obstacle.

## Challenges for state of health estimation and forecasting, remaining useful lifetime prediction in automotive applications

The tasks of SOH estimation and forecasting as well as RUL prediction, just as other algorithms and battery simulation models in the context of BMS and battery cloud solutions, face two major challenging domain shifts (DS): from laboratory or test bench operation to real-world BEV operation (DS1) and from LIB cell-level to LIB system-level (DS2) including the combination of both limitations (Cell & Lab → System & BEV).

First, battery aging in laboratory conditions differs significantly from battery aging in real-world BEV operation (DS1) as argued by^43,49,58–61^. In our previous work^18,19^, we argue that there is a continuous path towards laboratory data closer to real-world battery operation. Real-world battery operation includes idling, external charging, and driving as described in the taxonomy of battery operation in automotive applications in Supplementary Note 3. The main problem is that laboratory operation conditions are never equivalent to real-world BEV operation because they are meant to accelerate aging. Thus, it is necessary to get a deeper understanding of how real-world usage conditions affect battery aging and outline opportunities to approximate them better in the laboratory^62^. There is an ongoing effort on specifying more realistic laboratory tests, for example with variable discharge currents^63,64^ and combining cycling and calendar aging, i.e., both $I\,\ne\,0{A}$ and $I=0{A}$, in the same battery cell under test^65^. Calendar aging should also be examined in the laboratory with non-constant environment temperature like in real-world operation. This further extends to the importance of path dependency^65^.

Second, most of the batteries operated in laboratory are cells, but not modules or packs which are installed in BEVs (DS2). Aging of battery modules and systems is more complex than of a single cell because of the interactions of all cells. These interactions cause the degradation process of the battery pack and of the cells in that pack to depend on each other^66^. It is influenced by inconsistencies of cell characteristics, also known as intrinsic cell-to-cell variability^47^, electrical imbalance, and temperature gradients between cells that cause heat transfer to adjacent cells^45,46^. For data-driven methods, e.g., using machine learning, it is important to figure out how to apply them not only on cell level, but also on module-, pack- and system- level. This challenge becomes evident in two examples:

First, so far, electrochemical impedance spectroscopy (EIS) studies mostly examined individual cells or low voltage modules, with only a few studies dealing with HV battery modules and packs as required for BEVs^67,68^. Overall, EIS seems applicable to modules and packs with careful calibration of the model because the signals are harder to interpret for modules and packs^69^. Second, a virtual incremental capacity analysis (ICA) using a digital battery twin failed to yield conclusions regarding the SOH on system level, but was successful on cell level^38^.

## Operational battery data from vehicles: logging and aggregation

Logging operational data in mobile applications faces the challenge of limited and costly bandwidth and connectivity, when sending the logged data to a central backend like a cloud. Thus, the question of what data to log, as well as what and when to send to the cloud needs to be answered for BEVs as well. This concerns the measured controller area network (CAN) signals and their sampling time.

Recommendations on the measured signals and their sampling time to capture battery operation in BEVs are given in Table 1 depending on the dynamic of the respective signals. Voltage and current require the highest sampling rate because they are the most dynamic signals in automotive applications. Especially in driving mode in the wide sense, according to the taxonomy introduced in Supplementary Note 3, the current and voltage are not constant, but highly variable and non-linear due to the possibility of huge, but brief power demands^59,70–72^. For the temperature measurement during parking mode, it may be important to take regular measurements, e.g., every hour. This ensures capturing the dynamic of the environment temperature in the course of day and night. The capacity and storable energy content only change slowly with aging over time. Thus, a daily or weekly sampling should be sufficient. For all of the signals in Table 1, not only the sampling time, but also the measurement resolution and accuracy are relevant. They also may limit the benefit of a high sampling time. These benefits may be countered by the increasing cost for data transmission and data storage^61^.

**Table 1:** Recommendation for data logging of battery operation in automotive applications

Signal	Sampling time (see ref. ^13^)
*V*_cell_, *V*_pack_	100 ms
*I*_cell_, *I*_pack_	100 ms
*T*_cell_, *T*_pack_	On value change (1 s)
SOC	On value change (1 s)
Battery & vehicle mode (see Taxonomy in Supplementary Note 3)	On value change
Capacity, *SOH*_C_, *E*_max_	Daily/weekly

The sampling time is the inverse of the sampling frequency. “Logging” refers to the collection of data over time.

${V}_{{{\rm{cell}}}}$ and ${V}_{{{\rm{pack}}}}$ cell and pack voltage, ${I}_{{{\rm{cell}}}}$ and ${I}_{{{\rm{pack}}}}$ cell and pack current, ${T}_{{{\rm{cell}}}}$ and ${T}_{{{\rm{pack}}}}$ cell and pack temperature, *SOC* state of charge, ${{SOH}}_{{{\rm{C}}}}$ charged-based state of health, ${E}_{\max }$ maximum storable energy content.

However, data aggregation before sending operational data to the cloud may be advisable to reduce the data transfer cost. But also battery simulation models benefit from compact and representative load profiles as efficient inputs^43^. This especially makes sense, when battery data of long time periods, like weeks or months of operation, regarding battery aging is stored. Overall, there is a trade-off of data aggregation between information compression, data representativeness, human-interpretability, saving data-related costs for storage, processing, and transmission. The data aggregation method must align with the specific task and objective for which the data will be used. Currently, there are a few methods on encoding operational data densely in an aggregated form:

First, one-dimensional (1D) and multi-dimensional (2D, 3D) residence time histograms of the operational parameters current, SOC, and temperature have been used^19,73,74^. Color-coded, i.e., heatmap style, 1D and 2D histograms are still interpretable by engineers with some practice. Also, the order of the operational states is not encoded in the histograms but would matter due to path dependency of battery aging. However, the bin width and range of the histograms’ bins have to be chosen adequately and in advance, if the aggregation is executed in the vehicle.

Second, statistical properties of histograms like range, maximum, minimum, mean, variance, skewness, and kurtosis have also been used to aggregate the histograms further^75^. Zhang et al.^75^ argue that these statistical properties of histograms may encode the correlation of battery operation and aging better than the histograms. However, the interpretability for humans may be more complex and require a understanding of these descriptive statistical properties (e.g., see Fig. 2 in ref. ^75^). For example, an increase of the kurtosis of the SOC-histogram corresponds to an increase of residence time in the SOC states in the distribution tails (less peakedness, i.e., light-tailed), i.e., more time is spend SOCs distant to the mean SOC which usually means in low and high SOCs. Especially, the statistical properties of multi-dimensional histograms will likely become complex to interpret because of the multidimensionality of the statistical properties. Furthermore, they are not uniquely defined^76^.

Third, these properties could also be directly derived from the battery operational time-series data as in Table 5 in ref. ^77^. Here, likewise understanding of the descriptive statistical properties is necessary to interpret them correctly.

Fourth, operational patterns of the battery could be clustered unsupervised to aggregate data. Therefore two variants regarding the temporal size of clusters exist: one cluster's weekly operational patterns^78^ and another cluster's driving trips^79^. For both, if the aggregation is executed in the vehicle, the clusters need to be determined beforehand. In general, the task of determining suitable clusters has probably many feasible results. Beneficially, the clusters have characteristics that are easy to interpret, also for BEV users, like “charging on Thursday night”^78^ or “charge-every-night”^78^. Also, the representativeness of the identified patterns needs to be ensured.

The temporal splitting into weeks or trips of the last category can be combined with the histograms from the first category: temporal sub-sequences can be generated before the aggregation by histograms. This has the advantage to maintain some information about the sequence of operation compared to pure histograms: first, the most simple approach is to aggregate the battery operation monthly, weekly, daily, or per trip as suggested in our previous work^13^ (see Chapter 10.1 there). For example, the battery operation of each week could be described by separate histograms. Second, the rainflow algorithm can alternatively be used to separate operational sequences, combined with aggregation by histograms^80,81^. Rainflow counting enables the consideration of micro-cycles.

Even though the SOC will not likely change significantly during parking, the temperature of the battery certainly will depend on day–night, seasonal, shade–sunlight, and indoor–outdoor temperature differences which influence calendar battery aging. The temperature can either be approximated based on temperature data from public weather services or logged during regular wake-ups by the vehicle. It is likely that there are times during parking where it is not economically viable to log all data.

Apart from unsuitable and limited logged data, the progress on research tasks like SOC estimation, SOH estimation, RUL prediction, and SOH forecasting is also hindered by lacking comparability of the models’ performance^18,49,82^: exemplarily for SOH forecasting different metrics, different output values, and different forecast horizons are used^13,18^. A standardization of these criteria is probably difficult to achieve given the absence of a worldwide institution and different requirements of different battery applications, most likely, if at all, United Nations Economic Commission for Europe (UNECE) or International Standards Organization. Thus currently, comparability can only be ensured by using multiple metrics, output values, and forecast horizons so that a common overlap over several methods in this regard can be achieved.

Currently, no standard data set from real-world operation exists for battery SOH forecasting models like ImageNet, MNIST, or CIFAR for image classification models (see overview Table 12 in ref. ^19^). Such open-source datasets give different researchers the opportunity to compare the performance results, e.g., of their data augmentation techniques^83^. A publicly available data set not from laboratory battery operation, but from real-world vehicle operation of battery systems would enable benchmarking of SOH forecasting models applicable in real-world vehicle operation battery systems. Thus, it would accelerate research progress.

## Conclusion

This comment re-evaluates the current state of battery data and the SOH. The following suggestions for the development of the field, especially for automotive applications, were formulated:

- An SOH definition merging several scalars to a vector format was motivated to fulfill the Markov property. It may lead to more accuracy of SOH estimation and SOH forecasting methods, while the relative capacity (${{SOH}}_{{{\rm{C}}}}$) may still be practically relevant customers and fleet managers.
- Battery-cell classification after cell production might be diversified by extending the current ordinal grading system of battery cells into groups A, B, and C, potentially related to the previously proposed vector-based SOH. Also, the benefits of using data from battery manufacturing beyond cell production have been discussed.
- To extend research on SOH estimation, SOH forecasting, and RUL prediction from battery cell & laboratory operation to battery systems & BEV operation, battery operational data is differentiated and examples are provided.
- Battery data logging in automotive applications was discussed and methods for time-series data aggregation from battery operation like histograms, and statistical features including combinations with the generation of temporal sub-sequences were presented.

This paper focuses on the perspective on automotive applications, as mentioned in the title. Other applications like LIBs for grid storage are not so much in focus. Furthermore, passenger vehicles as automotive application are more in the foreground in this paper than trucks. Battery electric trucks (BETs) may have different characteristics regarding the battery compared to passenger vehicle: different usage, i.e., different battery load, different battery system configuration and different charging system, i.e., megawatt charging system (MCS) in the case of BETs.

A detailed discussion of the problem of transferring and scaling research results from universities to the scale of the automotive industry is still open^84^. Most universities conduct research on cell materials in small lab scale (often coin format) but automotive cell formats and larger cell sizes behave differently (often big prismatic or cylindrical cells). Furthermore, the scale-up of cell chemistry is expensive and difficult due to the altering of the thermal and mechanical behavior of battery cells when scaling. Additionally, automotive cell production needs to based on mass-scalable manufacturing technology, whereas cells from laboratory production can be handcrafted and individually optimized.

Overall, we observe an increasing research focus from battery cells operated in the laboratory towards battery systems in real-world applications that are only scratching the tip of the iceberg of the inherent complexity in battery systems in real-world applications. Especially, once more operational battery data from BEVs become available in the near future because of the rising adoption of BEVs in the vehicle markets research progress should accelerate.

## Supplementary information

Supplementary Information

## Data Availability

All data analyzed during this study are included in this published article and in the references.
