# Supplementary material for: The future of battery data and the state of health of lithium-ion batteries in automotive applications
Source: Commun Eng. 2024 Nov 19;3:173. doi: 10.1038/s44172-024-00299-w (PMC11577075; doi:10.1038/s44172-024-00299-w)
Supplement: Supplementary file 1 — Supplementary Information [file 44172_2024_299_MOESM1_ESM.pdf]

The Future of Battery Data and the State of Health of Lithium-ion Batteries in Automotive Applications

Friedrich von Bülow[a*], Felix Heinrich[b], and William Arthur Paxton[c]

[a] *Volkswagen AG, Berliner Ring 2, 38440 Wolfsburg, Germany;*
friedrich.von.buelow@volkswagen.de

[b] *Volkswagen AG, Center of Excellence Battery, 38231 Salzgitter, Germany*

[c] *Volkswagen Group of America Inc., Innovation and Engineering Center California, 500 Clipper Drive, Belmont, CA 94002, USA*

ORCID: Friedrich von Bülow 0000-0002-4355-2636; Felix Heinrich 0000-0002-4275-1481; William A. Paxton 0000-0001-5899-9038

The results, opinions and conclusions expressed in this publication are not necessarily those of Volkswagen Aktiengesellschaft.

Supplementary Information

Supplementary Notes 1: Overview of further SOH_R-definitions

There are also other definitions of the SOH_R such as $\frac{R(t)-R_\mathrm{nom}}{R_\mathrm{nom}}$ by[1], $\frac{R_\mathrm{EOL}-R(t)}{R_\mathrm{EOL}-R_\mathrm{nom}}$ by [2–4], and $2-\frac{R(t)}{R_\mathrm{nom}}=\frac{2\cdot R_\mathrm{nom}-R(t)}{R_\mathrm{nom}}$ by [5,6]. All SOH_R definitions start at 100 %. Some require the definition of an end-of-life (EOL) criterion R_EOL setting R_EOL as $SOH_\mathrm{R}=0$ % reference point. This has the advantage to be coherent with the SOH definitions of SOH_C and SOH_E which decrease for progressing aging, i.e., increasing R. The SOH_R of other definitions increases for progressing aging so that the term State of Aging (SOA) would be useful to distinguish the different viewpoint of these definitions.

Supplementary Notes 2: Definition of Arithmetic, Geometric, and Harmonic Means

Given a set of data points $x_1, \dots, x_n$, the following definitions apply:

For the arithmetic mean:[7]

$$\bar{\mathrm{x}}_{\mathrm{arithm}} = \frac{1}{\mathrm{n}} \cdot \sum_{i=1}^{n} x_i = \frac{x_1 + x_2 + \cdots + x_n}{\mathrm{n}} \quad (1)$$

where $\bar{\mathrm{x}}_{\mathrm{arithm}}$ is the best predicate because it minimizes the sum of $(x_i - \bar{\mathrm{x}})^2$. The arithmetic mean is suitable when x_i have the same units.

For the geometric mean:[7]

$$\bar{\mathrm{x}}_{\mathrm{geom}} = \left(\prod_{i=1}^{n} x_i \right)^{\frac{1}{n}} = \sqrt[n]{x_1 \cdot x_2 \cdot \ldots \cdot x_n} \quad (2)$$

which is suitable for x_i with different units of measure. The geometric mean is suitable when x_i have different units.

For the harmonic mean:[8]

$$\bar{\mathrm{x}}_{\mathrm{harm}} = \frac{n}{\frac{1}{x_1} + \frac{1}{x_2} + \cdots + \frac{1}{x_n}} \quad (3)$$

which is suitable for rates, i.e., for a ratio of variables with different measures. For example, the F_1-score is the harmonic mean of precision and recall[9,10]. The harmonic mean is high, only if all x_i are similarly high. I.e., a very low x_i cannot be compensated by another high x_i as easily as in $\bar{\mathrm{x}}_{\mathrm{arithm}}$ and $\bar{\mathrm{x}}_{\mathrm{geom}}$ (see **Supplementary Figure 1**).

For non-negative real x_i it holds:[11]

$$\min(x_1, \dots, x_n) \leq \bar{\mathrm{x}}_{\mathrm{harm}} \leq \bar{\mathrm{x}}_{\mathrm{geom}} \leq \bar{\mathrm{x}}_{\mathrm{arithm}} \leq \max(x_1, \dots, x_n) \quad (4)$$

as also visualized in **Supplementary Figure 1**.

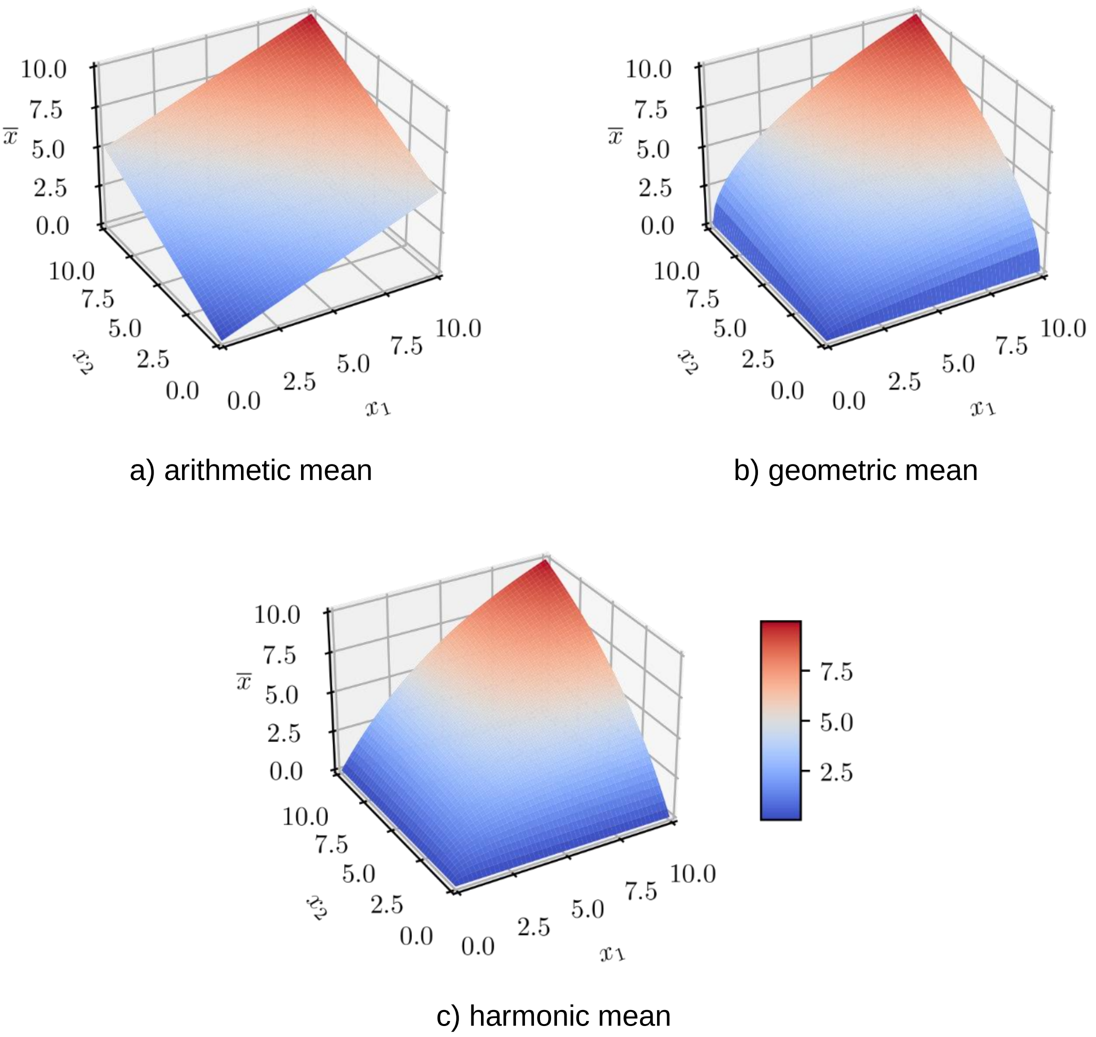

Supplementary Figure 1: **Arithmetic mean, geometric mean, and harmonic mean**

Graphical comparison of mean ($\overline{x}$) with two dimensions (x_1, x_2): **(a)** arithmetic mean, **(b)** geometric mean, and **(c)** harmonic mean.

Supplementary Notes 3: Taxonomy of Battery Operation in Automotive Applications

We differentiate the following operation modes of high-voltage (HV) batteries in automotive applications. The main criterion of the taxonomy is the current applied to the HV battery. The three classes are: $I = 0\ A$, $I < 0\ A$ for discharging, and $I > 0\ A$ for charging following the sign convention of International Union of Pure and Applied Chemistry (IUPAC)[12]. Further criteria are whether the power source for charging is BEV-internal (brake energy recuperation) or BEV-external in the form of charging infrastructure. For external charging the magnitude of the current and the use of either the BEV-internal or BEV-external AC-DC converter can be distinguished. These criteria are also used in standards as summarized in[13].

1. Idling/ resting/ pause/ parking ($I = 0\ A$ or $I \cong 0\ A$)
2. Charging with BEV-external power source ($I > 0\ A$), further distinguishable by, e.g.:
   a. AC-charging (using internal AC-DC-converter) and DC-charging (using external AC-DC-converter)
   b. Charging Levels from SAE J1772[14] or Charging Modes from IEC 61851[15] as summarized in[13].
3. Driving (in the wide sense):
   a. Recuperation, i.e., charging with BEV-internal power source using brake energy ($I > 0\ A$)
   b. Driving in the narrow sense/ discharging ($I < 0\ A$)
   c. Coasting ($I \approx 0\ A$): nothing is slowing down the BEV except air resistance and small friction from the moving parts.

References

1. Hu, X. *et al.* State estimation for advanced battery management. Key challenges and future trends. *Renewable and Sustain. Energy Rev.* **114,** 109334; 10.1016/j.rser.2019.109334 (2019).

2. Chen, L., Lü, Z., Lin, W., Li, J. & Pan, H. A new state-of-health estimation method for lithium-ion batteries through the intrinsic relationship between ohmic internal resistance and capacity. *Meausrement* **116,** 586–595; 10.1016/j.measurement.2017.11.016 (2018).

3. Noura, N., Boulon, L. & Jemeï, S. A Review of Battery State of Health Estimation Methods. Hybrid Electric Vehicle Challenges. *WEVJ* **11,** 66; 10.3390/wevj11040066 (2020).

4. Yao, L. *et al.* A Review of Lithium-Ion Battery State of Health Estimation and Prediction Methods. *WEVJ* **12,** 113; 10.3390/wevj12030113 (2021).

5. Ramadan, M. N. *et al.* Comparative Study Between Internal Ohmic Resistance and Capacity for Battery State of Health Estimation. *J. Mechatron. Electr. Power Veh. Technol.* **6,** 113–122; 10.14203/j.mev.2015.v6.113-122 (2015).

6. Remmlinger, J., Buchholz, M., Soczka-Guth, T. & Dietmayer, K. On-board state-of-health monitoring of lithium-ion batteries using linear parameter-varying models. *J. of Power Sources* **239,** 689–695; 10.1016/j.jpowsour.2012.11.102 (2013).

7. Grossman, J., Katz, R. & Grossman, M. *Averages: A New Approach* (Archimedes Foundation, 1983).

8. Ferger, W. F. The Nature and Use of the Harmonic Mean. *Journal of the American Statistical Association* **26,** 36–40; 10.1080/01621459.1931.10503148 (1931).

9. Goodfellow, I., Bengio, Y. & Courville, A. *Deep learning* (The MIT Press, Cambridge, MS, US, 2017).

10. F1-Measure. In *Encyclopedia of Machine Learning,* edited by C. Sammut & G. I. Webb. 1st ed. (Springer US, Boston, MA, US, 2010), p. 397.

11. Sedrakyan, H. & Sedrakyan, N. *Algebraic Inequalities.* 1st ed. (Springer International Publishing; Imprint: Springer, Cham, 2018).

12. Renner, T. Tables of physical quantities. In *Quantities, units and symbols in physical chemistry,* edited by E. R. Cohen*, et al.* 3rd ed. (IUPAC & RSC Publishing, Cambridge, 2008), pp. 11–248.

13. Rachid, A. *et al.* Electric Vehicle Charging Systems. Comprehensive Review. *Energies* **16,** 255; 10.3390/en16010255 (2023).

14. SAE International. *SAE Electric Vehicle and Plug in Hybrid Electric Vehicle Conductive Charge Coupler.* **J1772**. Available at https://www.sae.org/standards/content/j1772_201710/ (2017).

15. IEC. *Electric vehicle conductive charging system - Part 1: General requirements.* **61851-1:2017**. Available at https://webstore.iec.ch/publication/33644 (2017).
